# A parametric numerical study for the processing of highly reactive thermoset resins for liquid moulding applications

**DOI:** 10.1177/00219983251370393

**Published:** 2025-08-28

**Authors:** Leonardo Barcenas, Loleï Khoun, Pascal Hubert

**Affiliations:** 1 Department of Mechanical Engineering, 5620 McGill University, Montreal, QC, Canada; 2 CREPEC - Research Center for High Performance Polymer and Composite Systems, Montreal, QC, Canada; 3 National Research Council Canada, Boucherville, QC, Canada

**Keywords:** high reactive thermosets, thermomechanical behaviour, stress deformation, liquid moulding process

## Abstract

This study explores part geometrical deviations with manufacturing strategies for composite materials, focusing on highly reactive thermoset resins processed through Resin Transfer Moulding (RTM). A simulation framework that integrates the filling stage and stress-deformation analysis using a thermo-viscoelastic (TVE) model was developed to improve the understanding of material behaviour and its impact on part quality. The influence of key process parameters, including process temperature, nominal injection pressure, number of plies, and ply stacking sequence, was investigated for part geometrical deviations. The results show that the ply stacking sequence and the number of plies are the most significant factors affecting part geometrical deviation. In contrast, process temperature and injection pressure had only a minor effect. This work demonstrates the potential of the proposed simulation approach as a reliable tool for guiding experimental implementation and improving part quality when using highly reactive thermosets.

## Graphical abstract

**Graphical Abstract:**
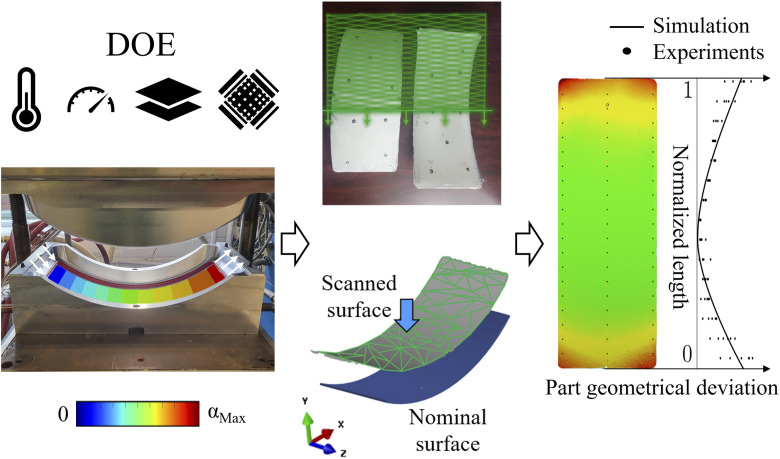

## Highlights

• Design of experiments to study the influence of process and lay-up parameters.
• Simulation framework accounting for degree-of-cure evolution during filling.
• Composite parts fabricated to validate the simulation predictions.
• Development of process maps to guide the optimization of parameters.
• Resin reactivity is more critical for impregnation than for stress-deformation.

## Introduction

Composite materials, characterized by their exceptional strength-to-weight ratio and versatility, have emerged as key components in various industrial applications, from aerospace to automotive engineering. However, achieving the required part geometrical accuracy during the manufacturing with these materials can be challenging due to the dimensional deviations caused by inherent residual stresses.^1–7^ Zobeiry et al.^8^ summarized the sources of residual stresses during the curing process of the matrix, which is a critical stage in the fabrication of composite structures. These stresses result from the following physical changes during cure: the cure shrinkage, the coefficient of thermal expansion (CTE), the mechanical properties including elastic modulus and Poisson’s ratio. Layup orientation, interactions between the composite part and the mould, and mismatches in CTE are also important factors contributing to the development of residual stresses.

To predict process-induced stresses and deformations, process modelling has become a common approach. Computational tools are implemented using the material models of the composite to compute the physical changes of the resin during the process as a function of defined process conditions such as temperature and mechanical boundary conditions.^1,8–12^ These analyses are used to predict the quality and properties of the final manufactured part, leading to a significant reduction in time and cost to manufacture the actual components.^13^ Accurate process modelling of composite manufacturing requires the integration of detailed and validated material models that represent the evolving behaviour of the resin throughout the curing cycle. These models should account for cure kinetics, the progression of mechanical properties such as the elastic modulus and Poisson’s ratio, as well as thermal and chemical shrinkage. Such accuracy is essential for the reliable prediction of temperature distribution, degree of cure, and the development of residual stresses during processing.^14–16^ This requirement becomes particularly important when working with highly reactive thermoset resins, which are widely used in the automotive industry to support large scale production of composite components. Manufacturing processes such as Resin Transfer Moulding (RTM), Compression Resin Transfer Moulding (C-RTM), and High-Pressure Resin Transfer Moulding (HP-RTM)^17,18^ rely on these fast-curing systems. The fast polymerization behaviour of these resins tends to affect the development of residual stresses during processing.^11,19^ Therefore, it is essential to apply robust and experimentally validated material models to achieve accurate simulations, optimize process conditions, and ensure the performance and dimensional quality of the final parts.

Barcenas et al.^20^ showcased the resin characterization of highly reactive thermosets (cure kinetics, viscosity, shrinkage, CTE, and thermo-elastic properties) with the combination of process simulations using PAM-RTM for the injection and Abaqus/COMPRO for the curing and stress analysis.^11,15,19–21^ The Cure Hardening Instantaneously Linear Elastic (CHILE) and the Thermoviscoelastic (TVE) models were used to study the elastic behaviour of Gurit Prime 130 Standard. Simulations indicated that the non-uniform degree-of-cure along the flow front during injection significantly influenced the evolution of matrix properties and the development of residual stresses. The CHILE model overestimated deformations due to its simplified assumptions and limited capability to capture the viscoelastic behaviour of the material, although it offers greater computational efficiency.^14,22^ In contrast, the TVE model considers the viscoelastic nature of the material through frequency and time domain master curves and provides more accurate predictions, though it requires higher computational resources.^22–24^ Experimental [0°/90°]_7_ composite parts were manufactured by injecting the resin at room temperature into a mould preheated to a constant temperature of 80°C. The nominal injection pressure was varied between 2 and 5 bar, which produced different degree-of-cure distributions along the flow front, with the highest values near the vent and the lowest near the injection port. A 38% variation was observed between the parts with maximum and minimum part geometrical deviations, emphasizing the critical role of process parameters and model selection in determining final part geometry.

Parametric studies are often conducted to explore the impact of various process parameters on the geometric stability of composite parts. Factors such as cure cycle, stacking sequence, and elastic modulus model were investigated for an L-shape geometry in Refs. 25 and 26. Findings indicated that TVE models were more accurate for complex parts requiring viscoelastic behaviour of the matrix. Critical parameters identified affecting spring-in and deformation response included the cure cycle and part thickness. L-shape versus U-shape geometries were also examined, with U-shape parts showing smaller bending moments but exhibiting web warpage, leading to larger spring-in compared to L-shape parts.^27^ Alternatively, studies on anti-symmetric laminates using CHILE and TVE models revealed higher accuracy in stress and deformation predictions with the TVE model, with the coefficient of friction showing minimal influence on residual stresses.^28^ Investigations into fast curing resins for V-shaped parts indicated that process temperature had the greatest impact on final part geometry, followed by lay-up, while cooling rate showed negligible influence on spring-in.^29^

While prior studies have highlighted the influence of cure cycles, part geometry, and material behaviour on the dimensional stability of composite structures, most of these investigations have focused on standard resin systems characterized by relatively slow curing kinetics. Consequently, their findings offer limited applicability to highly reactive thermosets. In the study that addresses highly reactive resin systems,^29^ the material behaviour was represented using the simplified CHILE model. Building upon the work of Barcenas et al.,^20^ who previously investigated the influence of degree-of-cure variations as a direct source of part geometrical deviation, the present study extends this line of research by incorporating those variations within the process simulations. Rather than isolating degree-of-cure as an independent factor, this work integrates its effects into a thermoviscoelastic material model to evaluate how such coupling improves the accuracy of deformation predictions under different processing conditions. To this end, this paper encompasses a two-level fractional factorial design of experiments with the purpose of identifying the main sources of part geometrical deviations among the manufacturing process parameters such as temperature, nominal injection pressure (P_nominal_), number of plies and stacking sequence. The investigation explores the relationship between cure kinetics, thermomechanical properties, number of plies, stacking sequence, and the resulting deformation behaviour, offering a foundation for advancements in the field of highly reactive thermosets processing.

## Process modelling and parametric study

### Process simulation

**Figure 1.**
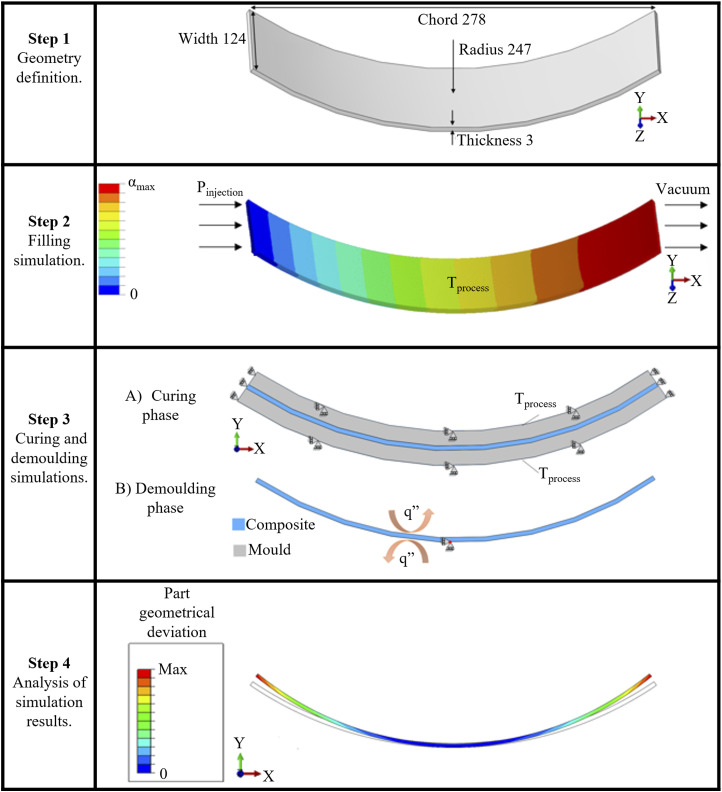
Description of the process simulation workflow. In Step 1, the geometry is defined. In Step 2, the boundary conditions for the filling simulation were set using PAM-RTM. In Step 3, the boundary conditions for the curing phase (a) and the demoulding phase (b) were defined using Abaqus. Step 4 involves analyzing the part geometrical deviation from the simulations, defined as the Y-component of displacement.

The process simulation was based on well-established physical models to accurately capture resin flow, heat transfer, viscosity evolution, cure kinetics, cure shrinkage, thermal expansion and modulus development. Detailed description of these models can be found in previous characterization and simulation work.^11,19–21,30–33^ Figure 1 summarizes the workflow for the process simulation strategy as defined in Ref. 20. The geometry of interest, defined in Figure 1, Step 1, consisted of a curved plate of 247 mm radius, 294 mm length, 124 mm width, and a thickness of 3 mm. The process simulation integrated the filling simulation for the injection stage with the stress-deformation simulation for the curing and cooling stages.

PAM-RTM software^34^ was used for the 3D filling simulation of heated resin transfer moulding. Tetrahedral (C3D4) elements were used for the composite part, with a total number of 7230 elements. Figure 1, Step 2 shows the boundary conditions used for the filling simulations, where the part surfaces were maintained at a constant mould temperature (*T*_process_), the resin was injected at room temperature (25°C) with a corrected injection pressure (*P*_injection_), and vacuum was applied on the opposite side of the injection point. The pressure implemented in the computational analysis was the corrected experimental nominal pressure, adjusted to account for losses due to tubing and flow front effects, based on the analysis by Deléglise et al.^35^

Abaqus^36^ and COMPRO^37^ software were used for the 2D stress-deformation analysis, which included both curing and demoulding phases. A thermal simulation was performed to calculate the evolution of temperature and degree-of-cure. An uncoupled mechanical simulation was followed to determine the stress-deformation based on the temperature and degree-of-cure from the thermal simulation. Figure 1, Step 3 outlines the thermal and mechanical boundary conditions for the curing and demoulding phases. An isothermal cure cycle was set at *T*_process_ and held for 30 min. The part was then released from the mould and cooled at room temperature by natural convection (heat transfer coefficient of 80 W/(m^2^K)). The stress-deformation simulations were conducted for a cross-section of the part under 2D plane strain conditions. A friction coefficient of 0.15 was defined between the mould and the part, with hard contact normal behaviour.^38^ C3D20 quadratic hexahedral elements were used for both the mould and the part in this 3D cross-section model. The part geometrical deviations, defined as the Y-component of displacement relative to the nominal part, were assessed at the end of the demoulding phase, as illustrated in Figure 1, Step 4.

### Material models

Gurit provided the Standard Prime 130 highly reactive thermoset resin system for this study. The cure kinetics model was previously characterized and modelled in Refs. 30 and 31. The coefficient of thermal expansion (CTE) and the cure shrinkage were reported in Refs. 11 and 31. The elastic modulus with the thermoviscoelastic (TVE) model was reported in Refs. 20 and 31.

E-glass non-crimp fabric from Texonic (TG-15-N NCS E-Glass) was used as reinforcement for this analysis. This fabric has an areal weight of 518 g/m^2^ and a warp/weft weight ratio of 44%–56%. The properties for E-Glass were obtained from Ref. 39, considering the elastic modulus E_E-Glass_ = 82 GPa, the shear modulus G_E-Glass_ = 30.13 GPa and the Poisson’s ratio ν = 0.22. Density, specific heat, coefficient of thermal expansion and thermal conductivity for E-Glass were obtained from the COMPRO database.^40–44^ Sarojini-Narayana et al. reported the permeability model for this preform.^19,32^ The COMPRO database was used to define the material properties of the mould, which was made of aluminum 6060. The material properties of the mould consisted of density, elastic modulus, Poisson’s ratio, specific heat, coefficient of thermal expansion and thermal conductivity.

### Experimental setup

**Figure 2.**
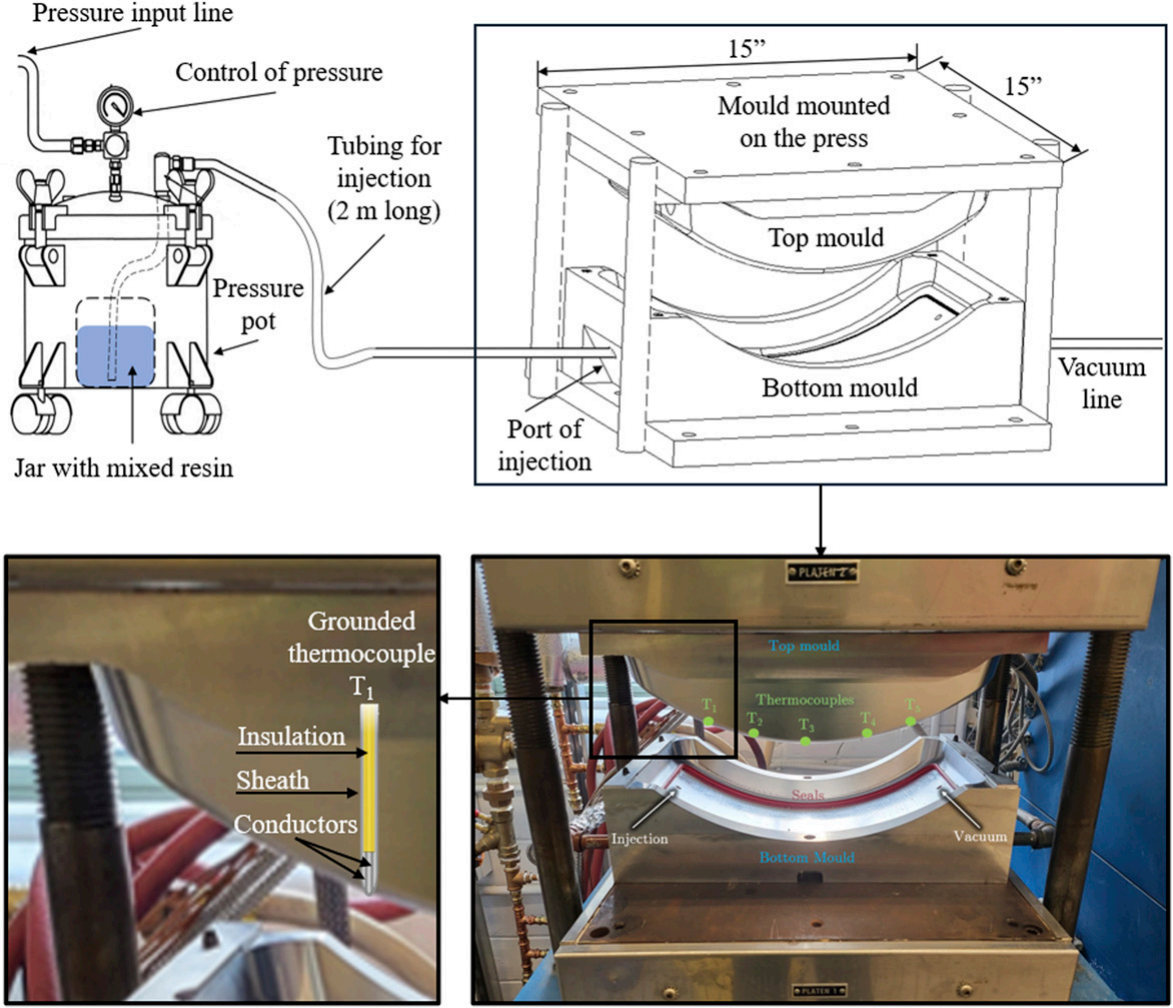
Schematic representation of the experimental setup. The fibre preform was placed into the mould at ambient temperature, after which the mould was fixed in the press and heated to the target processing temperature. Once the desired temperature was reached, the press was closed to initiate resin injection. The resin was injected at room temperature using a pressure pot set to the desired nominal pressure.

A mould was designed to reproduce the parts defined in Figure 1, Step 1. Computerized numerical control (CNC) machining was used to manufacture the mould made of aluminum 6060. Figure 2 illustrates the schematic configuration of the experimental mould setup. Four grounded K-type thermocouples were vertically embedded in the central region of the upper mould, with their tips in direct contact with the preform to monitor resin flow during the filling process. Mushroom-shaped seals were installed along the perimeter of the mould to ensure an effective seal. The fibre preform was manually cut and stacked in the mould outside the press under ambient conditions. The mould, containing the dry preform, was subsequently placed into a Wabash Genesis press, and the system was heated to the designated processing temperature. Once thermal equilibrium was reached, the resin was prepared and mixed at room temperature, then it was transferred into a pressure pot, where the nominal injection pressure was regulated using a control valve. The resin was injected through a 2-m-long tube with an inner diameter of 3.175 mm. Vacuum was applied to the outlet side to assist resin flow.

**Figure 3.**
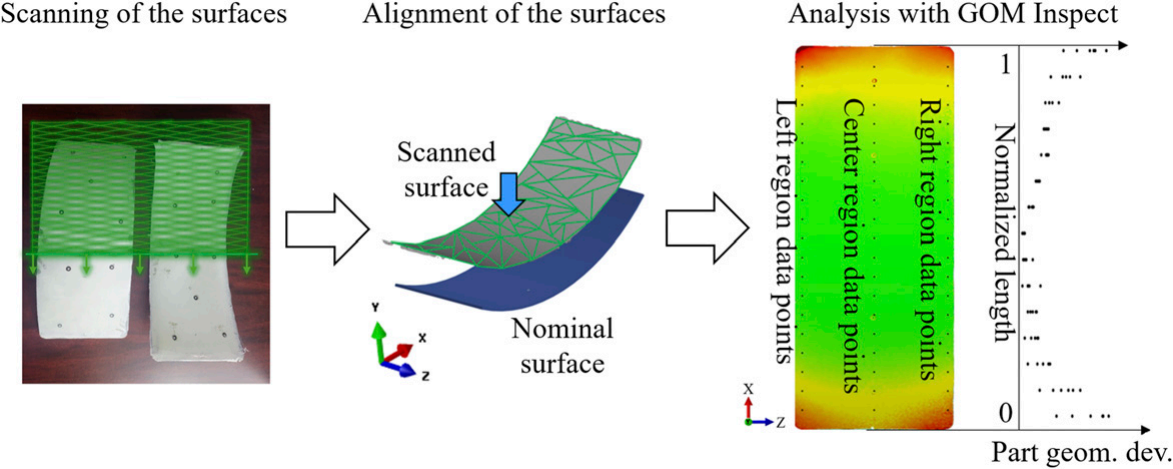
Sequence for acquiring the surfaces from the experimental parts using digital 3D scanning was conducted, involving a comparison with the simulation results using GOM Inspect software. Three regions of data points are defined along the normalized length of the part.

Figure 3 shows the sequence for 3D scanning and processing of the experimental parts. The surfaces of the experimental part were digitized using a GOM ATOS 5 3D scanner. The digitized part surfaces were aligned with the corresponding digitized mould surface (nominal surface). The part geometrical deviation was then processed using GOM Inspect software^45^ to measure the Y-component deviation. Three sets of data points were extracted from the left, centre, and right regions of the part for further comparison with the simulation results, as seen in Figure 3.

### Design of experiments

A two-level fractional factorial design of experiments was conducted to identify the main sources of part geometrical deviation. Table 1 shows the factors considered for this analysis with the low and high levels. The two-level fractional factorial design was carried out with Minitab Statistical Software. The experimental design was produced for the four factors into one block with eight runs with resolution IV, and the design generator D = ABC. The main effects are aliased with a three-way interaction such: A + BCD, B + ACD, C + ABD, and D + ABC. Two-way interactions are aliased with other two-way interactions, such: AB + CD, AC + BD, and AD + BC. Numerical process simulations were conducted for every configuration, and they were subsequently validated through experimental manufacturing.

**Table 1.** Processing factors with the two levels.

Factor designation	Factor name	Low level	High level
A	Temperature (°C)	80	100
B	Nominal injection pressure (bar)	4	5
C	Number of plies	5	7
D	Stacking sequence	[0/90]_N_	[+45/−45]_N_

The summary of the design is presented in Table 2, with the process parameters being process temperature (80°C–100°C) for 30 min, nominal injection pressure (4 bar–5 bar) at which the resin is injected at room temperature, number of plies (5–7), and stacking sequence ([0/90]_N_ - [+45/−45]_N_), where N denotes the number of plies. The response for the process simulation was the maximum part geometrical deviation, while for the manufactured components, it was defined as the average of 10 measured points with the highest part geometrical deviations.

**Table 2.** Summary of the two-level fractional factorial design of experiments with the maximum part geometrical deviation as response for both the experiments and the simulations. The initial α_min_–α_max_ is the degree-of-cure variations from the start (α_min_) to the end (α_max_) of the filling process simulation, as seen in Figure 1 step 2.

Experiment ID	Treatment	Factor A Process temp. (°C)		Factor B Nominal injection pressure (bar)		Factor C No. of plies		Factor D Stacking sequence		Initial α_min_–α_max_ for stress-deformation simulation	Response Maximum part geometrical deviation (µm)	
		Level	Value	Level	Value	Level	Value	Level	Value		Experimental	Simulation
T80P4N5_0/90	(1)	−	80	−	4	−	5	−	[0/90]_5_	0.01–0.09	1074	1532
T100P4N5_+45/−45	a	+	100	−	4	−	5	+	[+45/−45]_5_	0.01–0.14	1109	787
T80P5N5_+45/−45	b	−	80	+	5	−	5	+	[+45/−45]_5_	0.01–0.06	672	606
T100P5N5_0/90	ab	+	100	+	5	−	5	−	[0/90]_5_	0.01–0.11	1335	1920
T80P4N7_+45/−45	c	−	80	−	4	+	7	+	[+45/−45]_7_	0.01–0.14	386	374
T100P4N7_0/90	ac	+	100	−	4	+	7	−	[0/90]_7_	0.01–0.20	921	1127
T80P5N7_0/90	bc	−	80	+	5	+	7	−	[0/90]_7_	0.01–0.12	891	898
T100P5N7_+45/−45	abc	+	100	+	5	+	7	+	[+45/−45]_7_	0.01–0.18	556	485

Table 3 presents a summary of the regression model fitting for both the experimental and simulation results. The high coefficient of determination (R^2^) values, 93% for the experimental data and 91% for the simulations, indicate a strong correlation between the selected process parameters and the observed part geometrical deviations. These values confirm that the models capture most of the variability in the response, thereby demonstrating a high level of predictive reliability for both the physical trials and the numerical simulation.

**Table 3.** Summary of model performance for experimental and simulation results. Reported metrics include the standard deviation of residuals (S), the coefficient of determination (R-sq), the adjusted R-sq accounting for the number of predictors and observations, and the predicted R-sq (R-sq (pred)), which estimates predictive capability through cross-validation.

	S	R-sq	R-sq (adj)	R-sq (pred)
Experimental	129	92.7%	82.9%	47.8%
Simulation	199	91.0%	86.1%	57.5%

## Results and discussion

**Figure 4.**
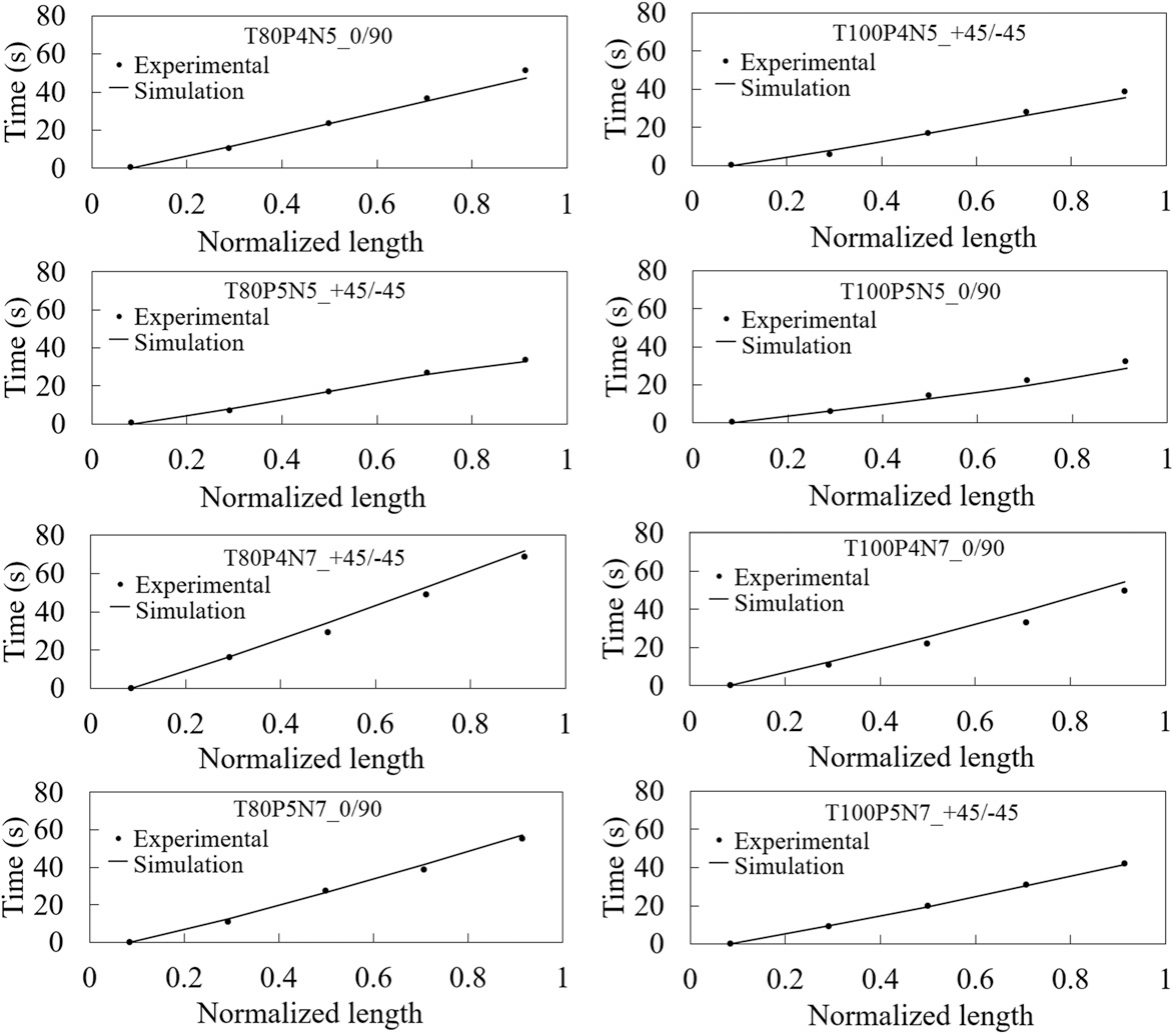
Comparison between the flow front measured with the thermocouples and the simulations on PAM-RTM with the corrected injection pressure for the different experimental configurations.

Figure 4 compares experimental flow tracking with filling simulations across the experiments. The simulations accurately predicted the resin flow front evolution for all cases. The longest filling time occurred with 4 bar injection, 80°C mould temperature, seven plies, and a +45/−45 stacking sequence, while the shortest was with 5 bar, 100°C, five plies, and a 0/+90 sequence. Results confirm that higher fibre volume fractions reduce permeability and slow flow, while increased injection pressure and mould temperature enhance flow rate by boosting pressure-driven flow and reducing resin viscosity.

**Figure 5.**
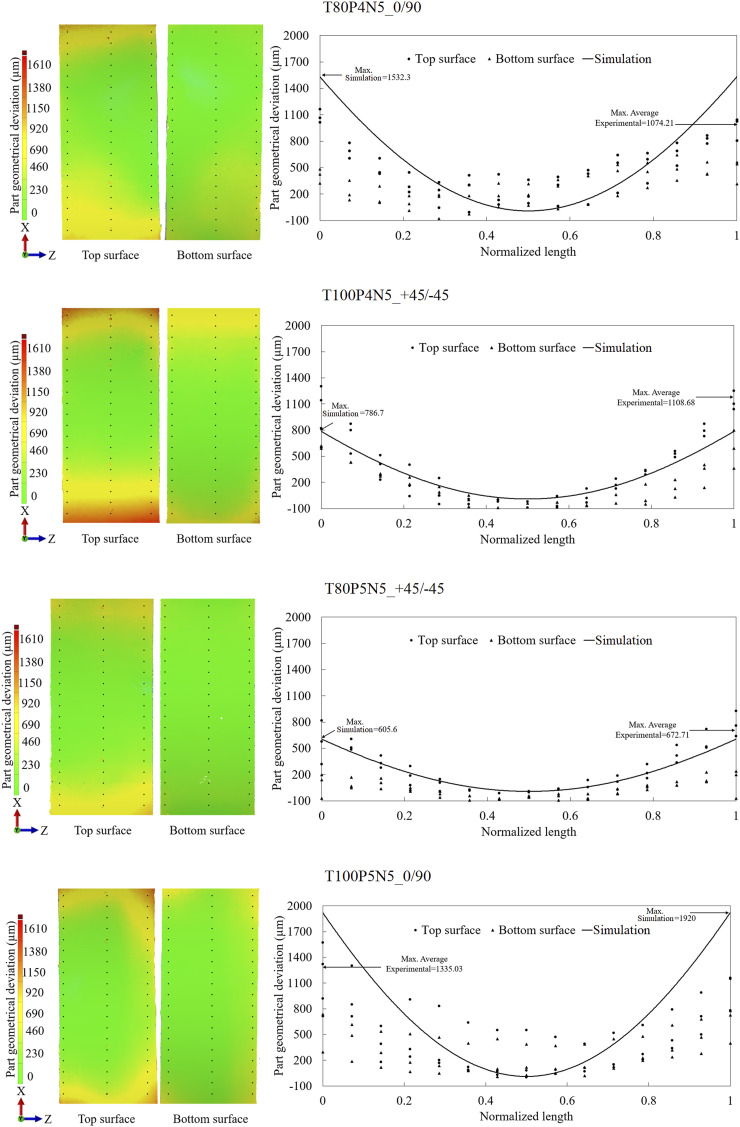
Experimental part geometrical deviation for experiments with five plies: T80P4N5_0/90, T100P4N5_+45/−45, T80P5N5_+45/−45, and T100P5N5_0/90, where points along the part on X direction were taken to plot them and compare with the simulation results.

**Figure 6.**
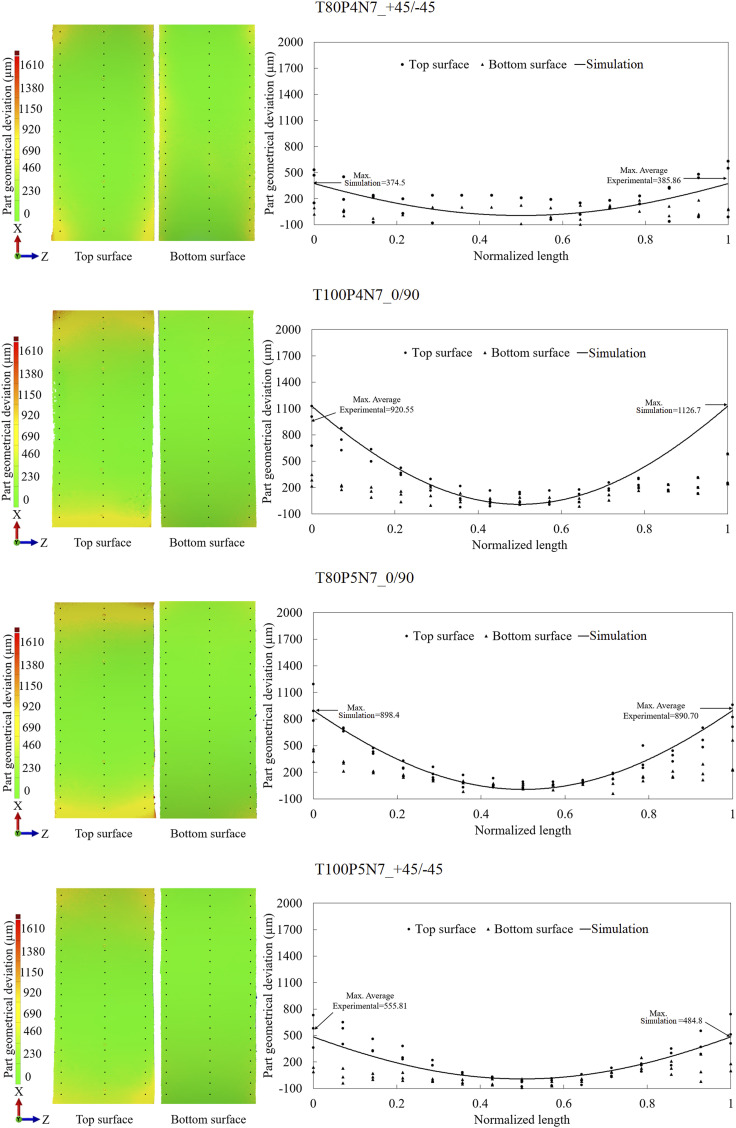
Experimental part geometrical deviation for experiments with seven plies: T80P4N7_+45/−45, T100P4N7_0/90, T80P5N7_0/90, and T100P5N7_+45/−45, where points along the part on X direction were taken to plot them and compare with the simulation results.

The detailed analyses of the 3D surface scans obtained from the experimental parts were divided into Figures 5 and 6. The left images illustrate the top and bottom surface contours. The right plots show the geometrical deviations, extracted at specific positions along the part length, for the simulation and experimental measurements.

Simulation results for cases T100P4N5_+45/−45, T80P5N5_+45/−45, T80P4N7_+45/−45, T80P5N7_0/90, and T100P5N7_+45/−45 fall within the scatter of the experimental data, indicating high predictive performance. However, numerical deviations for cases T80P4N5_0/90, T100P5N5_0/90, and T100P4N7_0/90 deviate significantly from the experimental measurements, predicting much higher geometrical deviations. The common characteristic among the latter cases is the stacking sequence of [0/90]_N_. This suggests a higher sensitivity of the simulation model to fibre orientation, and further investigation into the influence of processing parameters on dimensional accuracy was performed.

**Figure 7.**
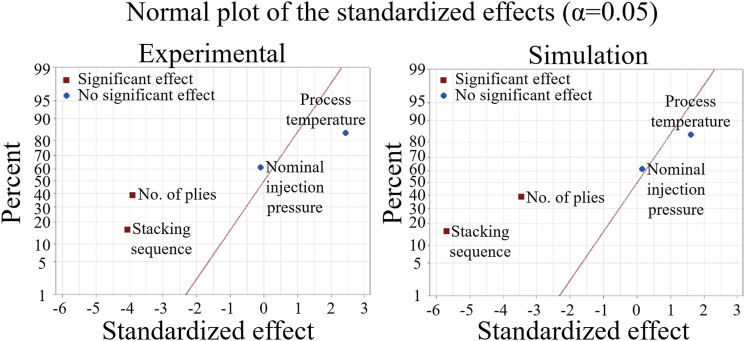
Normal plot of the standardized effects for the experimental parts and the simulation results from Figure 5. The maximum part geometrical deviation as the response.

Figure 7 complements the surface contour analysis by presenting the normal probability plots of the standardized effects derived from the two-level fractional factorial design. The response variable is the maximum part geometrical deviation. The normal probability plots evaluate the statistical significance of each parameter under the assumption of no effect (zero baseline). Both the experimental and simulation datasets reveal that the nominal pressure of injection causes minimal influence on final geometry, with effects located near the zero line. Processing temperature exhibits a higher impact, with deviations increasing when the temperature rises from 80°C to 100°C. Despite these variations, the magnitude of temperature effects remains statistically less significant when compared to more dominant parameters. In both experiments and simulations, the number of plies and stacking sequence emerge as the most influential factors affecting part geometrical deviation. Increasing the number of plies from 5 to 7 and modifying the stacking sequence from [0/90]_N_ to [+45/−45]_N_, reduces the part geometrical deviation. The number of plies contributes a standardized effect of −4.0 (experiments) and −3.5 (simulations), indicating consistent trends. Finally, the stacking sequence shows a more pronounced effect in the simulations (−5.5) than in experiments (−4.0), confirming the model’s sensitivity to these parameters, as observed in Figures 5 and 6.

**Figure 8.**
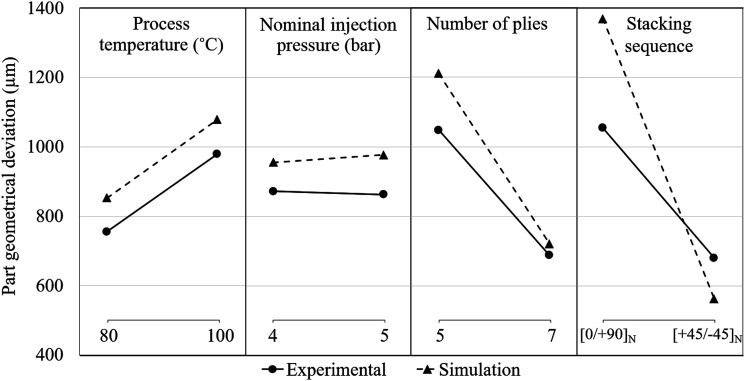
Main effects of process parameters with maximum part geometrical deviation as response for the experimental results and the simulations.

Figure 8 presents the main effects of the tested parameters on part geometrical deviation. Each plot displays the mean response at each level of a categorical variable, with lines connecting the means to highlight trends. The increase in part deviation due to temperature rise from 80°C to 100°C is nearly identical in both datasets (224 µm experimental vs 227 µm simulated). For the nominal pressure of injection when varying from 4 to 5 bar, experimental results show a negligible reduction in part deviation (−9 µm), while simulations indicate a minor increase (22 µm). In contrast, the number of plies has a significant effect when changing from 5 to 7 plies, with a decrease in part deviation of 360 µm in experiments versus a decrease of 490 µm in simulations. Despite this, simulations with seven plies demonstrate better agreement with experimental results than those with five plies.

For the stacking sequence, a reduction in part deviation of 374 µm (experiments) and 806 µm (simulations) is observed when switching from [0/90]_N_ to [+45/−45]_N_, reaffirming the sensitivity of the simulations to this parameter.

Barcenas^31^ attributes these discrepancies with experiments to several physical phenomena that are not accounted for in the simulations. These include the generation of micro-voids during curing,^46,47^ preform washout during injection,^48^ and fibre misalignment from manual handling and fluid-induced displacement during the filling.

**Figure 9.**
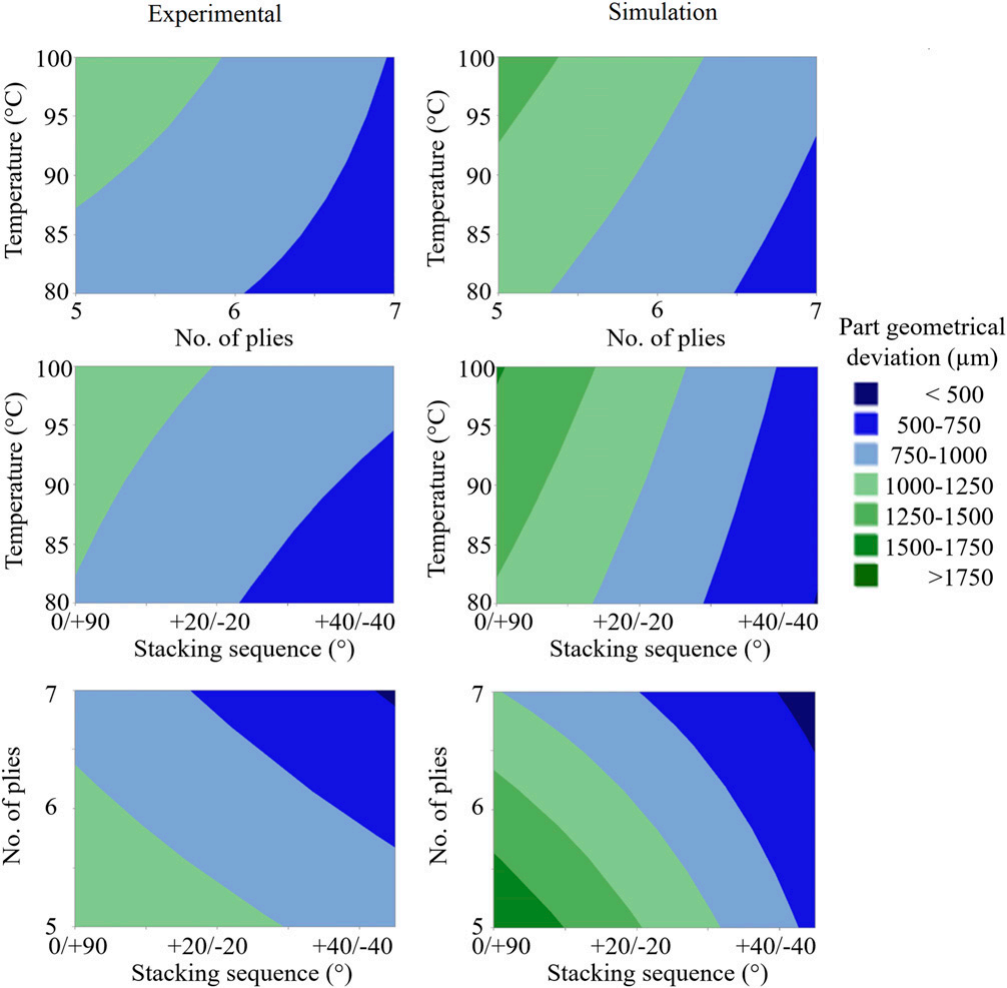
Process parameters map with maximum part geometrical deviation as the response obtained from Minitab software. Comparison of the experimental and simulation results.

Figure 9 presents a comparison of the process maps generated from both the experimental results and the simulation data. Process maps are particularly useful for selecting appropriate processing parameters during the filling stage of manufacturing with highly reactive thermosets^35^ to ensure a full impregnation of the preform within the available processing window before the resin reaches its gel point. To evaluate the influence of processing parameters on dimensional stability, process maps were generated using Minitab, with the part geometrical deviation defined as the primary response. These maps were constructed to identify regions of minimized part geometrical deviation as a function of key process variables. The contour plots were derived from the fitted regression models and focused on the three most influential parameters, such as the process temperature, number of plies, and stacking sequence.

Minitab implement the regression models to estimate response surfaces and generate two-dimensional contour plots. Each plot displays the relationship between two of the selected parameters while holding the remaining variables constant at predetermined neutral values derived from the experimental design (specifically: process temperature of 90°C, nominal injection pressure of 4.5 bar, six plies, and a [+22.5/−22.5]_N_ stacking sequence). The generated regions are confined within the defined experimental bounds, i.e., between the high and low levels of each parameter.

The first comparison, showing the number of plies versus the process temperature, reveals that both experimental and simulation results favour high ply count and low temperature for minimizing part geometrical deviation. However, the simulations predict higher maximum part geometrical deviation (1250–1500 µm) compared to the experiments (1000–1250 µm), indicating a more conservative model behaviour.

The second map, presenting the stacking sequence versus the process temperature, shows that simulations are largely insensitive to temperature with the staking sequence range of [+40/−40]_N_ to [+45/−45]_N_, whereas experiments suggest an influence of both factors. Again, simulations predict a higher part geometrical deviation envelope in the worst-case region.

The final map, showing the stacking sequence versus the number of plies, highlights a clear agreement, where minimal part geometrical deviations are achieved with [+45/−45]_N_ stacking sequence and seven plies, while the worst outcomes occur with [0/90]_N_ and five plies. Yet, simulations exhibit heightened sensitivity, predicting deviations as high as 1750 µm compared to 1250 µm experimentally. This reinforces the prior conclusion that the simulation model overestimates the effect of stacking sequence, particularly in low-plies configurations.

## Conclusions

This study investigated the influence of critical process parameters on the part geometrical deviation of composite components manufactured using highly reactive thermosets. The simulation framework coupled the resin transfer moulding (RTM) process with the subsequent curing and cooling stages, implementing a thermo-viscoelastic (TVE) model to predict deformation behaviour.

The results demonstrated that structural design parameters, particularly the number of plies and the stacking sequence, exert the most significant influence on the final shape of the manufactured part. The parametric analysis demonstrated that configurations with a higher number of plies and stacking sequence, such as [+45/−45]_N_, resulted in reduced part geometrical deviation. These results are consistent with previous research,^49–52^ which also identified stacking sequence and fibre volume as key factors in controlling part geometry. These findings underscore the critical need to align the structural design of composite components with their manufacturing process. Specifically, the number of plies and stacking sequence selected to meet mechanical performance criteria must also consider their impact on dimensional stability. It is important to note that this study focuses on thin-walled components, where temperature gradients and resin flow behavior are relatively uniform. The results may not directly apply to thicker parts, which can experience more pronounced deformation due to internal thermal and flow variations.

Additionally, this study incorporated the use of process maps, generated through simulation and validated with experimental results, to support decision-making in process optimization. These maps provided a visual framework for identifying favourable combinations of process parameters, offering reliable predictions for part quality. The results showed that the simulation-based maps were in good overall agreement with the experimental outcomes, demonstrating their potential as practical tools for experimental implementation and industrial application.

In conclusion, this study highlights an important aspect of composite manufacturing with highly reactive thermosets. While processing parameters such as injection pressure and process temperature are critical to achieve full impregnation, they do not have an important influence in controlling the part geometrical deviations. The results show that the stacking sequence and the preform volume fraction are the primary factors influencing part geometrical deviations, which aligns with observations from standard composite manufacturing.^8,12,25–28^ Nonetheless, process temperature effects are not negligible and should still be considered, especially in processes where small part geometrical deviations could be critical or where interactions with other parameters may amplify this role. The main challenge in using highly reactive thermosets is to ensure full impregnation before gelation occurs.^32,33,35^ Inadequate impregnation compromises both structural performance and manufacturing reliability. Through the integration of process simulations, experimental validation, and predictive process maps, this work presents a framework to support the selection of appropriate process conditions and preform configurations, enhancing manufacturing consistency and part quality in composite production using highly reactive thermoset systems.

## Data Availability

The data that support the findings on this study are available from the corresponding author, Pascal Hubert, upon reasonable request.*
